# The Fixation and Saccade P3

**DOI:** 10.1371/journal.pone.0048761

**Published:** 2012-11-07

**Authors:** Sangita Dandekar, Jian Ding, Claudio Privitera, Thom Carney, Stanley A. Klein

**Affiliations:** 1 Vision Science Graduate Program, University of California, Berkeley, California, United States of America; 2 Helen Wills Neuroscience Institute, University of California, Berkeley, California, United States of America; 3 School of Optometry, University of California, Berkeley, California, United States of America; 4 Department of Bioengineering, University of California, Berkeley, California, United States of America; University of Muenster, Germany

## Abstract

Although most instances of object recognition during natural viewing occur in the presence of saccades, the neural correlates of objection recognition have almost exclusively been examined during fixation. Recent studies have indicated that there are post-saccadic modulations of neural activity immediately following eye movement landing; however, whether post-saccadic modulations affect relatively late occurring cognitive components such as the P3 has not been explored. The P3 as conventionally measured at fixation is commonly used in brain computer interfaces, hence characterizing the post-saccadic P3 could aid in the development of improved brain computer interfaces that allow for eye movements. In this study, the P3 observed after saccadic landing was compared to the P3 measured at fixation. No significant differences in P3 start time, temporal persistence, or amplitude were found between fixation and saccade trials. Importantly, sensory neural responses canceled in the target minus distracter comparisons used to identify the P3. Our results indicate that relatively late occurring cognitive neural components such as the P3 are likely less sensitive to post saccadic modulations than sensory neural components and other neural activity occurring shortly after eye movement landing. Furthermore, due to the similarity of the fixation and saccade P3, we conclude that the P3 following saccadic landing could possibly be used as a viable signal in brain computer interfaces allowing for eye movements.

## Introduction

You are walking down the street when you notice someone waving at you in your peripheral vision. You make an eye movement in the direction of the waving and recognize a friend.

Scenarios such as the above, wherein a moment of visual recognition is preceded by a saccade, are quite commonplace. On average, humans make 2–3 saccades a second [1], [2] and natural vision is undoubtedly a dynamic, active process. However, most studies of the neural correlates of visual object recognition, and, more specifically of the P3 EEG signal, are conducted in situations in which the subject passively fixates while objects are presented at the fovea.

Herein, experiments are presented that attempt to equate visual stimulus characteristics in an object recognition task conducted at fixation and in an object recognition task requiring a saccade. It was hypothesized that the P3 observed after a saccade landing would differ in amplitude, latency and/or persistence from the fixation P3. This hypothesis was based on a variety of past work that demonstrated that visual processing is altered around the time of a saccade, with various past research observing modulations of neural responses and visual perception occurring prior to a saccade [3]–[5] and post-saccadic modulations of neural activity occurring after saccadic landing [4], [6]–[9]. Saccade related modulations in neural activity have been found to be both diverse and complex. For example, recordings in macaque MSTd hint at a complex interplay between modulations of evoked visual responses and modulations of background spontaneous firing rates in the time periods immediately before and after saccades [10]. Interestingly, in the presence of saccades, depending on the presence or absence of visual stimulation, both decreases in BOLD signal as well as increases in BOLD signal have been observed in human LGN and V1 [11]. Furthermore, various groups have hypothesized that object recognition and attention orienting could be enhanced after a saccade due to the known coupling of attention and saccades [12], [13]. Based on the post-saccadic modulations described in past studies, it was hypothesized that the saccadic P3 could be enhanced in amplitude and/or decreased in latency relative to the fixation P3.

Importantly, past electrophysiological evidence for post-saccadic modulation has mainly been quantified by directly comparing neural activity after a saccade to a baseline level of neural activity measured at fixation. Given that there are a multitude of neural processes that are thought to occur after a saccade but not during fixation (for example, the updating of gaze-centered coordinate systems [14] and the occipital saccadic lambda response [15], [16]), the functional role(s) of such post-saccadic modulations remain unclear. In contrast, herein, rather than comparing saccade and fixation neural data directly, we instead compared the difference of the target and distracter post-saccadic neural signals (the saccade P3) and the difference of the target and distracter signals measured at fixation (the fixation P3). Any general changes in neural activity due to the occurrence of a saccade (as well as the EOG artifacts known to co-occur with saccades in EEG) were therefore expected to cancel in the saccade target minus saccade distracter subtraction. Importantly, in addition, we equated the visual stimuli used for P3 targets and distracters [17], so that any post-saccadic modulations in sensory responses were expected to cancel out in the saccade target minus saccade distracter subtraction. It was therefore ensured that any differences that were observed between the fixation and saccade P3 could be disambiguated from post-saccadic modulations of sensory neural activity.

The P3 has been used by multiple research groups as the basis of brain computer interfaces [18], [19]. Therefore, in addition to determining whether or not the P3 is affected by post-saccadic modulations in neural activity, another main motivation for studying the P3 at saccade landing is that the saccadic P3 could be useful in the development of improved brain computer interfaces (BCIs) allowing for natural eye movements. Quantifying the characteristics of the saccadic P3, such as amplitude, persistence and start time, and, furthermore, quantitatively comparing saccadic and fixation P3 characteristics as is done here could provide useful information for use in the training of pattern classifiers used in BCIs [20], [21].

There have been a few observations of P3 activity following saccadic landing [22]–[24] but, to the best of our knowledge, only one prior study has attempted to directly compare the fixation and saccade P3 [25]. However, as will be further explained in the discussion, in the aforementioned study, target and distracter visual stimuli were not equated [17], thereby leading to post saccadic differences between saccade and fixation conditions that were likely sensory in origin rather than P3 differences.

Herein, when the saccade P3 and the fixation P3 were compared, it was determined that there were no significant differences in P3 start time, temporal persistence, or amplitude dependent on whether the P3 was measured at fixation or after a saccade. The similarity of the fixation and saccade P3 indicates that the saccadic P3 could be a viable signal for use in BCIs.

The lack of significant differences between the saccade and fixation P3s does *not* contradict past research that has found post-saccadic modulations of early sensory neural responses (see Discussion). The results do, however, indicate that post-saccadic modulations are likely limited to sensory and other neural processes occurring in the early stages (approximately first 200 ms) after saccade landing. We conclude that post-saccadic modulations in neural activity do not significantly affect the P3.

## Materials and Methods

### Subjects

Data were collected from eight subjects (four males and four females). All subjects signed informed consent forms prior to the start of experiments. The experiments were approved by the Committee for the Protection of Human Subjects at the University of California, Berkeley. All subjects ranged in age from 20–30 years, had normal vision without need of correction, and were right handed. All subjects except S1 and S6 were naïve in regard to the aims of the study.

### Eye Position and EEG Data

The Eyelink eyetracker was used to track horizontal and vertical eye position. Eye position was sampled at 1000Hz.

An Active Two EEG System (Biosemi) was used to collect EEG data simultaneously with eye movement recording. The 64 electrodes were used in a headcap with the standard Biosemi 64 electrode layout. Details pertaining to the method of referencing of the Biosemi system during data collection can be found at: http://www.biosemi.com/faq/cms&drl.htm. In addition to the 64 electrodes on the scalp, two electrodes were placed on the mastoids during data collection. After data collection, all scalp electrode data were referenced to the mean of the two mastoid electrodes. Electrode P04 was excluded from analyses for S1, S2 and S7 and electrodes T7 and TP8 were excluded from analyses for S3 because it was noted during data collection that the aforementioned electrodes were not maintaining good contact, likely due to localized looseness of the electrode cap. All 64 electrodes were retained for within subject analyses for the other subjects. In cross subject analyses, only the 61 electrodes that were retained for all subjects were analyzed.

Analog eye position signals from the Eyelink were fed into the Biosemi input box. In this manner, the horizontal and vertical eye positions recorded by the Eyelink were sampled along with the EEG at 512 Hz. The Biosemi system also recorded stimulus event markers generated by the stimulus presentation system (WinVis psychophysical and physiological testing toolbox).

### Target and Distracter Stimuli

**Figure 1:**
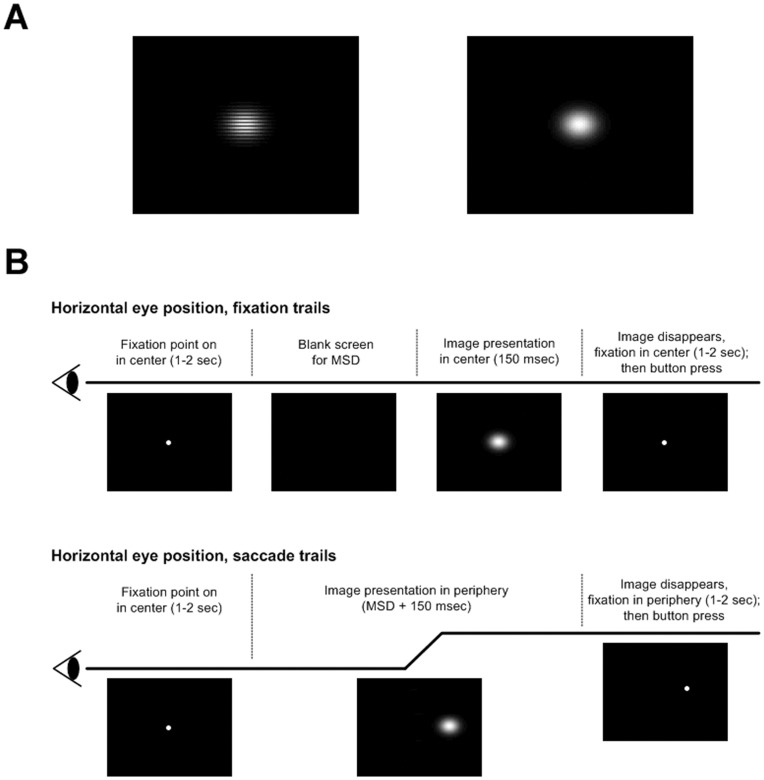
Saccade and fixation stimuli. **A**, Two stimuli that were interchangeably used as target and distracter stimuli. **B**, Time course of horizontal eye position in fixation and saccade trials and visual stimuli corresponding to each temporal interval. The mean saccadic delay (MSD) was estimated prior to image presentation for each subject. The blank frames in the fixation trials were set to appear for a duration equal to the MSD prior to target/distracter presentation of 150 ms. The duration of image presentation in saccade trials was set to 150 ms plus the MSD in order to ensure that the image presentation duration after saccadic landing was also approximately 150 ms.

The definition of target and distracter was switched in a counterbalanced manner from one block of 70 trials to the next to rule out differences in the target/distracter neural responses that could be due to low level differences in the target and distracter images [17]. One of the images that could be either a target or a distracter consisted of a 2-D Gaussian multiplied by a rectangular wave (25.5 cycles/degree) on a black background. For both saccade and central fixation trials, the targets/distracters were defined as being either the image shown in the left of Figure 1A or just a Gaussian pedestal without the rectangular wave addition (Figure 1A, right). The two stimuli were designated as either the target/distracter on an equal proportion of trials for all subjects. In other words, image 1 was defined as a target just as often as image 2 for every subject, and therefore image 1 and image 2 were also defined as distracters for the same number of trials for each subject. Trial numbers were equated before trial exclusion procedures for artifact rejection. Before each block of 70 trials, subjects were informed of which image would be defined as the target and which image would be defined as a distracter for that block of trials. The 2-D Gaussians used for both targets and distracters had horizontal and vertical standard deviations of 1°. For both saccade and fixation trials, 20% of the total images presented were targets.

Blocks of saccade and fixation trials were interleaved with randomized order of presentation. Prior to beginning each block of 70 trials, subjects were informed whether it would be a block of fixation or saccade trials. 560–840 trials were presented for each subject, with 50% of the trials being fixation trials, 25% involving leftward saccades, and 25% involving rightward saccades.

### Temporal Image Presentation Sequence

Subjects had to foveate a fixation point (diameter 1.2°) for a variable period of 1–2 seconds. In saccade trials the fixation dot disappeared and the target/distracter stimulus appeared unpredictably on either left or right side at 10° distance for 150 ms plus the mean saccadic delay (MSD) estimated individually for each subject. (See following section for details of MSD estimation). This ensured that each subject had approximately 150 ms to distinguish a target from a distracter after saccadic landing. Similarly, in fixation trials, after the variable length fixation period, the fixation point disappeared and the screen went blank for a duration of the estimated MSD prior to a 150 ms long target/distracter presentation in the fovea. In both the fixation and the saccade trials, target/distracter presentation in the fovea and the periphery, respectively were followed by a replacement of the target/distracter image with a fixation point centered at the previous location of the target/distracter.

Subjects were instructed to maintain fixation and delay their target versus distracter button press until the variable length fixation period (varying from 1 to 2 seconds) that followed target/distracter presentation ended (signified by disappearance of the fixation point). Target and distracter button presses were done with the same (right) hand. (Figure 1B).

### Procedures to Approximately Equate Fixation and Saccade Image Presentation Times

In order to compare the saccade and fixation P3, procedures were developed to minimize the difference between: 1) image presentation duration in the fixation trials and 2) the image presentation duration *after saccadic landing* in the saccade trials. In order to approximately equate image presentation duration in fixation trials and image presentation duration after saccadic landing in saccade trials, the MSD from when a target/distracter was presented in the periphery to saccadic landing was measured for each subject in a set of 70 trials during which only eye tracking data were recorded. The MSD was defined as the mean across the 70 image presentations in the periphery of the sum of the time to saccade onset after image presentation in the periphery (i.e. subject reaction time) plus the duration of the saccade before saccade landing (i.e. transit time from fixation to the peripheral target). Image presentation duration in fixation trials was 150 ms. The duration of image presentation in saccade trials was set to 150 ms plus the MSD as individually measured for each subject in order to ensure that the image presentation duration after saccadic landing was also approximately 150 ms.

### Image Visibility Control Procedures

Both target and distracter images had Michelson contrasts relative to the black background of 0.95. The overall contrast of the images was incremented/decremented randomly between + and –5% to remove any low frequency cues to stimulus identity that subjects might be able to distinguish using their peripheral vision prior to saccade onset. The random jittering was applied both to stimuli in the saccade condition and the fixation condition.

The presence or absence of the high spatial frequency rectangular grating that distinguished a target from a distracter was not detectable without making a saccade. This was validated for every subject by performing test trials prior to EEG data collection during which each subject maintained central fixation and attempted to distinguish targets from distracters without moving their eyes from central fixation when the targets/distracters were presented in the positions 10° from center as they would be in saccade trials. It was found that all subjects were performing at chance levels in the test trials. All of the subjects also verbally confirmed that they were unable to distinguish the target and distracter stimuli in the periphery without moving their eyes from the central fixation point.

### EEG and Eye Movement Pre-processing

EEG data were filtered with a low pass infinite impulse response (IIR) filter from the EEGlab toolbox [26] with a cutoff of 40 Hz and a high pass IIR filter with a cutoff of 0.3 Hz. Both the high and low pass filters had zero phase distortion due to forward and reverse filtering of the data. The EEG data from fixation trials were epoched using the data flanking one second (±1) from the moment of image presentation (time 0). Similarly, in the case of saccadic trials, epochs were extracted and event-related averages were calculated ±1 second relative to saccadic landing. Even with high pass filtering, saccades are expected to introduce step-like DC artifacts to EEG data due to the rotation of the cornea-retinal dipoles during saccades. Since the cornea retinal dipoles change positions during saccades, a different DC offset is expected before and after a sacccade [27], [28]. Therefore, in addition to the highpass filter, in both fixation and saccade trials, a baseline DC level for every fixation and saccade trial was determined by averaging the DC voltage offsets associated with a 200 ms long period located from −300 to −100 ms before the time locking event (the time locking event was image presentation time for fixation trials and saccade landing for saccade trials) and a 100 ms long period located from 900 ms to 1000 ms after a trial’s time locking event. The mean DC voltage as determined over these pre and post windows was subtracted from each fixation and saccade trial as a baseline estimate. Note that the saccade trials had two different DC offsets in the pre and post windows defined above due to the aforementioned effects of cornea retinal dipole rotation during saccades, whereas fixation trials tended to have more similar DC offsets in the pre and post windows. The same scheme was used for baselining for both trial types, however, in order to facilitate comparisons of the P3s observed in the fixation and saccade cases. The purpose of the aforementioned baselining procedures was to minimize the differences in DC offsets between trials prior to calculating EPs. Note that the baseline procedures did not remove step functions associated with saccades. As described in the following paragraph, saccade step artifacts were minimized in this study due to the equivalent number of leftward and rightward saccade trials.

When there are approximately an equal number of leftward and rightward saccade trials with approximately equal saccade amplitudes used to calculate a saccade-locked event related average, the DC step artifacts for leftward and rightward saccades tend to cancel in the event related average due to the leftward and rightward step artifacts having approximately equal amplitudes with opposite polarities [27]. In this study, there were an equal number of leftward and rightward saccade trials with the same saccade amplitudes. Since our primary purpose was to compare the saccade and fixation P3, we therefore averaged leftward and rightward saccadic P3 responses and did not attempt to address effects pertaining to lateralization of saccades on the P3.

For both trials at fixation and trials with saccades, epochs with saccades exceeding 1° following image presentation and saccade landing, respectively, were excluded from analyses. A previously described [29] microsaccade detection algorithm was used for detection of breaks from fixation.

### Identifying the P3 in Fixation and Saccade Trials within Subjects

Standard, non-parametric cluster statistics that are commonly applied to both fMRI [30] and EEG/MEG analyses [31], [32] were applied to test for significant differences between target and distracter trials in the evoked potentials (EPs) time locked to image presentation in the fixation trials and time locked to saccadic landing in the saccade trials. Cluster statistics measure the false alarm rate, account for the multiple comparisons problem, and take into account correlations across electrodes and across time. The non-parametric methods for statistical testing were applied using Fieldtrip software for EEG/MEG analysis (see http://www.ru.nl/fcdonders/fieldtrip, Donders Institute for Brain, Cognition and Behavior, Radbound University Nijmegen, the Netherlands) and are described briefly below.

**Figure 2:**
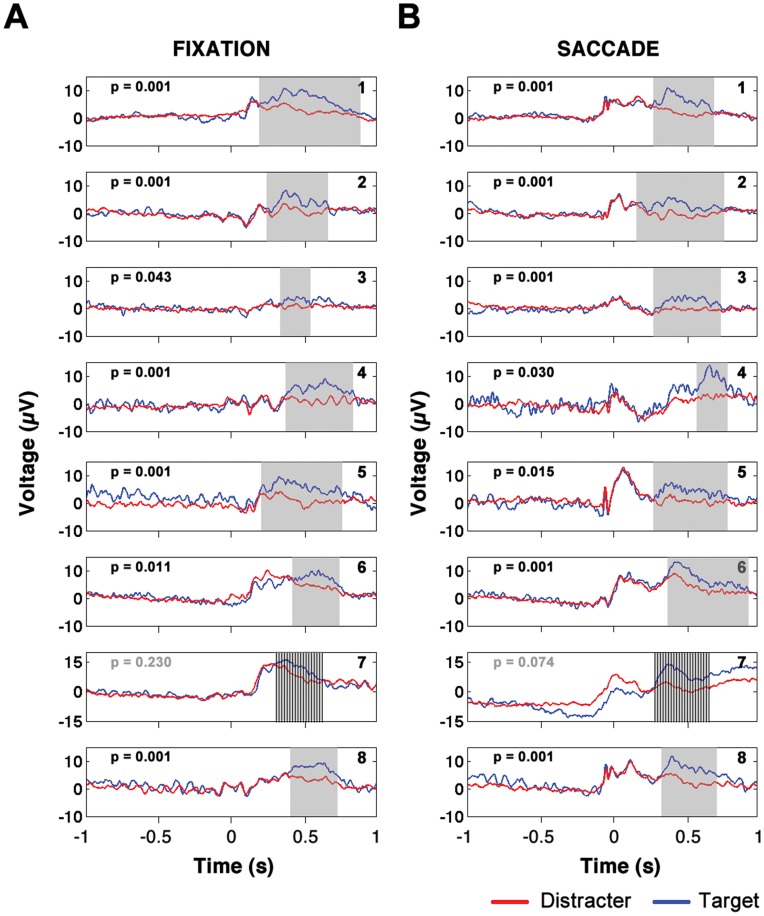
Individual subject saccade and fixation P3s. Plot of mean activity of electrodes in significant clusters of target minus distracter positivity for fixation trials (left column) and saccade trials (right column). Mean time course of target trials is plotted in blue and mean time course of distracter trials is plotted in red. Note that the EP waveforms are averages over all electrodes that showed significant target minus distracter positivity at any time points over the whole time window in which significant target minus distracter differences were observed. Time is measured relative to image presentation (fixation condition) or relative to saccade landing (saccade condition).

The first step in applying cluster statistics was to gather all of the target and distracter EEG epochs in a single condition (saccade or fixation) into a single pool of epochs. For each subject, if there were T target trials and D distracter trials, for each of 1000 iterations, T and D epochs were randomly selected from the pool of epochs of all types with disregard to whether the T and D randomly selected epochs were target or distracter trials. Target minus distracter t-statistics were determined for both the real target and distracter epochs and the 1000 iterations of randomly chosen target and distracter epochs. On each iteration, t-statistics were “clustered”, or summed across temporally adjacent time points and spatially adjacent electrodes displaying a single sample p-value of 0.05 or less. The maximum of the sum of the clustered t-statistics was recorded on each iteration, thereby forming a null distribution of t-statistic maxima observed in the randomly chosen epochs. The final p-value for any given cluster of target versus distracter difference observed in the real data could be determined by calculating the percentage of maxima of summed t-statistics in the null distribution greater than the t-statistic sum associated with the cluster observed in the real data. One-tailed tests using the null distributions calculated separately for saccade and fixation trials were used to identify significant clusters of target minus distracter positivity (P3 signals). One-tailed tests were used due to the well-known positivity of the P3 when referenced to the mastoids [33]–[35]. The results of analyses described in this section are shown in Figure 2.

### Comparing the P3 in Fixation and Saccade Trials within Single Subjects

**Figure 3:**
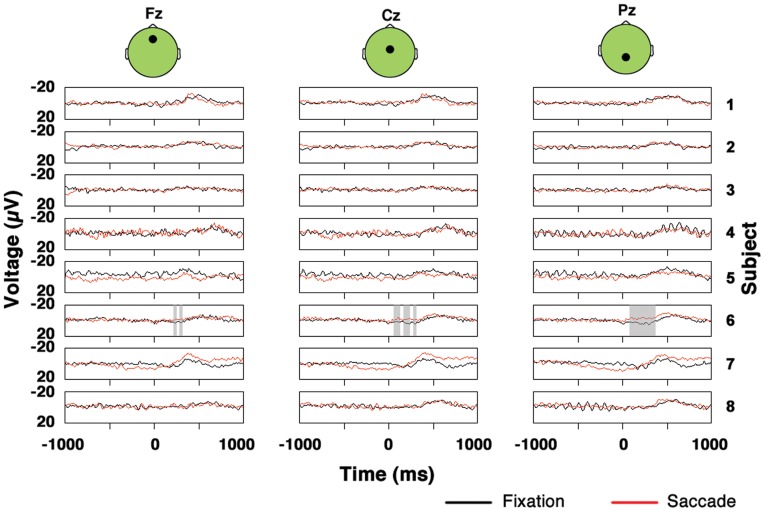
Saccade and fixation target minus distracter data at midline electrodes for individual subjects. Periods of significant difference between the saccade and fixation conditions are shaded.

Non-parametric methods similar to those described above were used to compare the fixation and saccade P3s observed within each subject separately. For all single trial target epochs observed in the fixation and saccade data, the mean distracter evoked potentials (for fixation and saccade trials, respectively) were subtracted from the target single trial epochs. The procedure resulted in a set of target epochs with mean distracter signal subtracted out for fixation trials and a comparable set of target epochs with mean distracter signal subtracted out for saccade trials. The saccade target epochs with mean distracter EPs subtracted and fixation target epochs with mean distracter EPs subtracted were tested for significant differences using cluster statistics in exactly the same way that the target and distracter epochs within a single condition were compared (cluster statistics application described in previous section.) However, the test on the null distribution was two-tailed rather than one-tailed. The results of analyses described in this section are shown in Figure 3.

### Comparing the P3 in Fixation and Saccade Trials in Cross Subject Average

In order to compare fixation and saccade P3s across subjects, as a first step, for each subject at every electrode and time point, the differences between target and distracter EP signals were expressed as t-statistics for both the fixation and saccade conditions. In order to account for inter-subject differences in SNR, the resulting t-statistic waveforms were converted into z-statistic waveforms before cross subject averaging. The procedure resulted in z-score waveforms for fixation trials and z-score waveforms for saccade trials for every subject, or 2N waveforms total (where N is the number of subjects, and there are 2 waveforms corresponding the 2 conditions per subject) at every electrode. The null hypothesis to be tested was that the target minus distracter (TMD) z-score waveforms in the saccade case were equivalent to the TMD z-score waveforms in the fixation case. Comparisons between the TMD EPs in the fixation and saccade conditions were performed using non-parametric cluster statistics similar to those described previously. A null distribution of differences between the saccade and fixation conditions expected due to chance alone was created by permuting the definitions of saccade and fixation TMD EPs within every subject independently and calculating the grand EP for every permutation. Note that the two conditions were interchanged only within subjects and not across subjects. The interchanging procedures therefore resulted in 2^N^ (where N is the number of subjects, and there are 2 conditions: saccade and fixation) values of the null distribution that could be compared to differences observed in the actual grand average EP across subjects. The value that was input into the null distribution for each permutation was the maximum of the sum of the t-statistics observed in the permuted grand average EP over clusters of adjacent electrodes and time points that each met a single sample significance of p<0.05. The other details of the cluster statistics were the same as discussed previously except the test against the null distribution to compare saccade and fixation TMD EPs was two-tailed.

**Figure 4:**
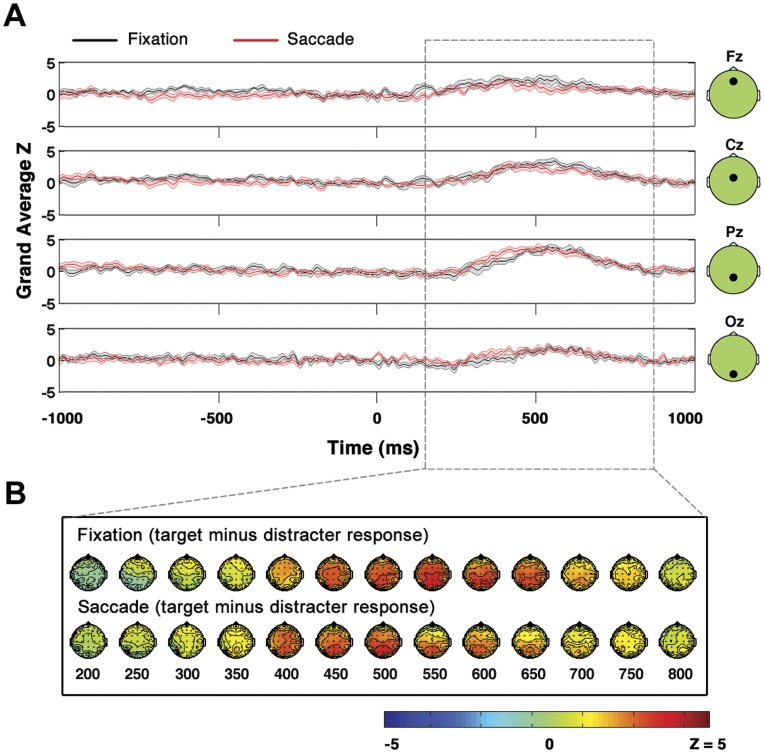
Comparison of fixation and saccade P3s. When P3s in saccade and fixation conditions are compared, saccade related EOG and EEG activity cancels in the saccade target minus saccade distracter subtraction, and there are no significant differences observed between saccade and fixation conditions. **A,** Grand average across subjects of P3 signal (target minus distracter) at midline electrodes Fz, Cz, Pz and Oz. Time is measured relative to image presentation (fixation condition) or relative to saccade landing (saccade condition). Shaded area represents standard error. **B,** Topographies of target minus distracter difference in saccade and fixation trials.

**Figure 5:**
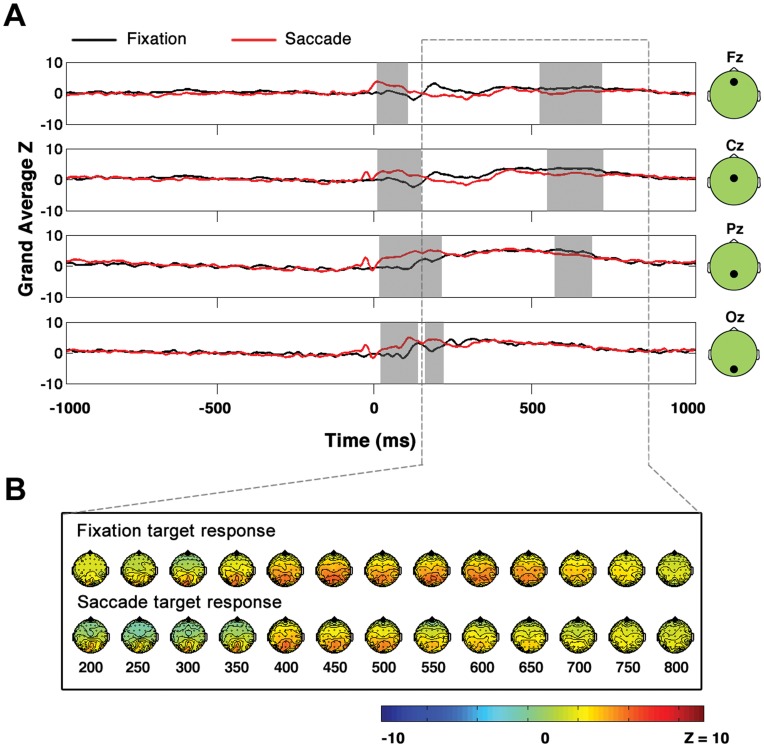
Direct comparison of fixation target and saccade target EPs. In contrast to figure 4, when saccade target and fixation target EPs are compared directly, significant differences are observed between the saccade and fixation conditions, likely due to EOG and EEG saccade-related activity present in the saccade but not the fixation condition**. A,** Grand average across subjects of fixation and saccade target responses at midline electrodes Fz, Cz, Pz and Oz. Time is measured relative to image presentation (fixation condition) or relative to saccade landing (saccade condition). Time points belonging to clusters of significant difference are highlighted in gray. **B,** Topographies of target responses in fixation and saccade trials.

**Figure 6:**
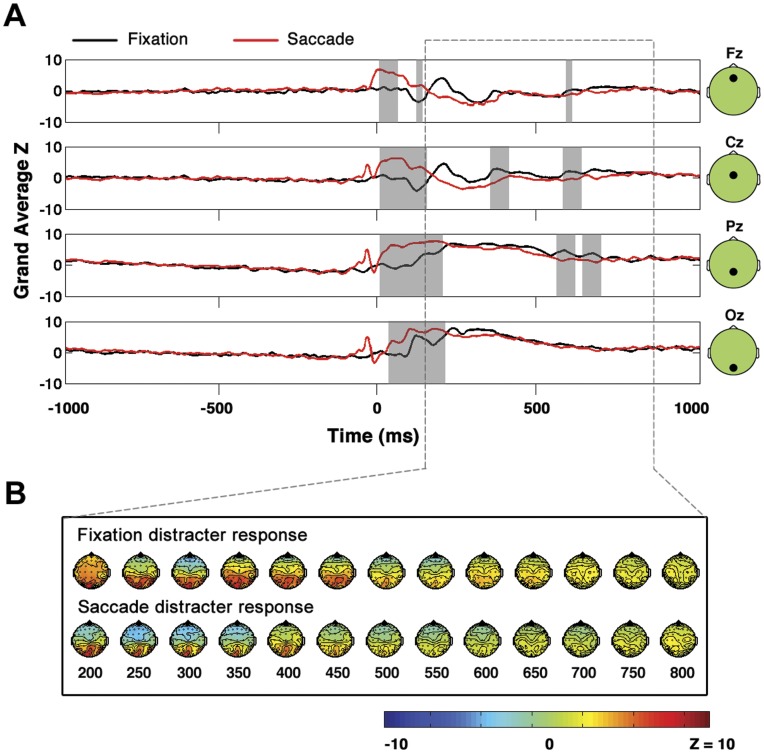
Direct comparison of fixation distracter and saccade distracter EPs. Same as Figure 5 but for distracters rather than targets.

Effect size (g) associated with differences between the grand average saccade and fixation P3s at any given time point were quantified via the following formula [36]:
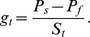
Where *P_s_* and *P_f_* are values of the saccade and fixation P3 target minus distracter EP waveforms, respectively, at the time point (t) at which the effect size is to be evaluated, and *S_t_* is the pooled standard deviation across the saccade and fixation conditions calculated using:



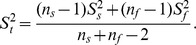
Where *n_s_* and *n_f_* are the number of observations of the saccade and fixation EP waveforms that contribute to the cross subject grand average (equal to the total number of subjects contributing to the cross subject grand average, in both cases). *S_s_* and *S_f_* are, respectively, the standard deviations across subjects of the saccade and fixation P3s at any given electrode and time point at which it is desired to estimate the effect size. The results of analyses described in this section are shown in Figures 4, 5 and 6.

### Quantifying Start Time and Persistence in Cross Subject Average

For every cross-subject grand average P3 (target minus distracter) z-score waveform, the total positive area under the P3 waveform was found via integration. Start times of the cross-subject grand average P3 waveforms were defined at each electrode as the time that it took for the positive area under the curve to rise to a level at which five percent of the total positive area under the curve was covered. Similarly, the end time of the P3 was defined as the time that it took for the positive area under the curve to rise to a level at which ninety-five percent of the total positive area under the curve was covered. Persistence was defined as the difference in time between the start time and the end time as defined above.

Non-parametric methods very similar to those described in *Comparing the P3 in fixation and saccade trials across subjects* were used to test the cross subject grand averages in the fixation and saccade conditions for differences in start time and persistence. After defining start time and persistence at each electrode in the grand average, the mean of each of these parameters across all electrodes was calculated. The difference between saccade and fixation for both parameters was calculated as the difference in the means across all electrodes as determined separately for the saccade and fixation conditions. To test the mean differences across all electrodes in start time and persistence for both parameters, a null distribution of the differences expected between saccade and fixation conditions due to chance alone was created by performing pair-wise permutations within subjects of the saccade and fixation waveforms (using the same permutation procedure described in *Comparing the P3 in fixation and saccade trials across subjects*). On each iteration, the grand average EPs of the randomly permuted data were calculated, and the mean difference between the two parameters of interest in the randomly permuted data were calculated, resulting in 2^N^ values of the null distribution. The actual differences in start time and persistence observed between the grand average saccade and fixation P3s could be tested against the start time and persistence null distributions generated as described above (two-tailed tests).

## Results

### Behavioral Results

The one second period after saccade landing in saccade trials and the one second period after image presentation in fixation trials were examined for microsaccades using a microsaccade detection algorithm [29]. Trials in which microsaccades exceeded 1° were excluded from both trial types. In addition, saccade trials were excluded if the saccadic delay on any given trial was longer than 300 ms, and also excluded if the saccadic delay on any given trial was below 100 ms or occurred prior to target presentation in the periphery. Furthermore, saccade trials were not included if eye movement data indicated that, on any given trial, the saccade from the central fixation point did not terminate on the target/distracter in the periphery. The above procedures resulted in retention of 89±2% of fixation trials and 75±6% of saccade trials on average across subjects (all results will be reported in the form of mean ± standard error).

During the 70 pre-experiment test trials to estimate the MSD for each subject, across subjects, the MSD was 240±6 ms. During the experimental EEG trials, the MSD across subjects was 213±6 ms. Therefore, on average across all subjects, the procedures to equate mean image presentation duration in the fixation condition and the saccade condition were able to minimize the difference in image presentation duration between conditions to 27±6 ms. Because the difference in image presentation time between saccade and fixation trials was significant (paired t-test; p = 0.003, t(7) = 4.57), the differences between P3 parameters in the saccade and fixation conditions observed for each subject (P3 amplitude, start time, and persistence) were tested for correlations with the differences in image presentation times between the saccade and fixation conditions observed for each subject. No significant correlations between the difference in image presentation time between saccade and fixation conditions and the corresponding differences in P3 parameters were found (See *Testing P3 parameters for dependence on the difference in image presentation time* for further details).

When subjects foveated the target and distracter stimuli, the two types of stimuli were designed to be very easily distinguishable, with subject button presses indicating that all subjects were correctly identifying targets and distracters with over 95% accuracy in both saccade and fixation trials. Note that the button presses were therefore included not for the purpose of behavioral analyses, but only to encourage subjects to remain as engaged as possible in the tasks. Any errors in subject button presses were therefore assumed to be manual or careless errors rather than difficulties in discrimination between target and distracter. The decision to design the target and distracter stimuli to be easily distinguishable was made due to the fact that the P3 signal’s amplitude has been shown to decrease if the target and distracter stimuli are not easily distinguishable [34], and it was desired to evoke a reliable P3 in both the fixation and saccade conditions.

### P3 in Fixation and Saccade Trials

In Figure 2, it can be seen that in all but one subject, both the central fixation trials and the saccade trials yielded significant clusters of positivity of target trials relative to distracter trials at the p = 0.05 level or less (one-tail cluster statistic). The one exception was subject 7, for whom the fixation task yielded a P3 with p = 0.23 and a saccade P3 with a false alarm probability of p = 0.074. Since no significant fixation or saccade P3s were observed in S7, this subject’s data were not included in cross subject analyses in which the fixation and saccadic P3 were compared. (It was noted during data collection that there was an abnormally large amount of low frequency drift artifact in S7’s EEG data, therefore resulting in large trial-to-trial variability. This low frequency drift was likely the reason that the fixation and saccade P3s did not reach significance in S7.).

The spatial topography of the P3 in both the saccade and the fixation case was widespread on the scalp. 62±1 electrodes belonged to significant clusters of P3 activity in the fixation condition and 59±2 electrodes belonged to significant clusters of P3 activity in the saccade condition on average across subjects.

The P3 start time for each subject (which was defined as the first time point at which any electrode showed a significant TMD difference) did not differ significantly: 312±38 ms for fixation trials and 321±46 ms for saccade trials (paired t-test across subjects; t(6) = −0.21, p = 0.84, two tailed).

Similarly, the persistence of the P3 (defined here as the temporal duration of the significant cluster of TMD positivity as determined across all electrodes) was found to not differ significantly between fixation and saccade trials across subjects (persistence in fixation trials 418±58 ms; persistence in saccade trials 468±45 ms; paired t-test; t(6) = −0.63, p = 0.55, two tailed).

At every time point at which there was a significant P3 signal (significance determined by application of non-parametric cluster statistics as described in Methods), the mean TMD positivity was calculated only over those electrodes that were significant at that particular time point. A mean over all of the significant time points was then calculated. The P3 amplitude across all subjects as defined above was 4.39±0.38 µV in fixation trials and 4.78±0.53 µV in saccade trials. The difference in the amplitude of the P3 between fixation and saccade trials was not significant across subjects (paired t-test; t(6) = −1.01, p = 0.35, two tailed).

P3 signal strength was also quantified for each subject by first expressing the mean TMD positivity at each time point and electrode as a t-statistic. Similar to the measure of P3 amplitude described above, at every time point at which there was a significant P3, the mean P3 amplitude was calculated over only those electrodes that exhibited a significant P3 difference at each time point. The signal strength was defined as the mean t-statistic as observed over all of the significant time points. The P3 signal strength did not differ significantly between the saccade and fixation conditions across subjects (mean P3 amplitude expressed as a t-statistic in fixation trials: 2.6±0.1; mean amplitude measure expressed as t-statistic in saccade trials: 2.5±0.1; t(6) = 0.85, p = 0.43, two-tailed).

### Testing P3 Parameters for Dependence on the Difference in Image Presentation Time

All parameters of the saccadic and fixation P3 that were compared across the two conditions were tested for correlations with the difference in image presentation time observed between the two conditions (see *Behavioral results)*. Recall that, on average across subjects, the difference image presentation time between saccade and fixation conditions was minimized to be 27±6 ms. Differences in image presentation time between the fixation and saccade conditions for each of the 7 subjects with significant saccade and fixation P3s were not significantly correlated with the corresponding differences in P3 amplitude, persistence, start time, or signal strength observed between the fixation and saccade conditions for each subject. (Difference in amplitude of P3 not significantly correlated with difference in image presentation time: r(5) = 0.45, p = 0.31; difference in start time of P3 not significantly correlated with difference in image presentation time: r(5) = 0.28, p = 0.54; difference in P3 persistence not significantly correlated with difference in image presentation time: r(5) = 0.50, p = 0.26; difference in P3 signal amplitude expressed as a t-statistic not significantly correlated with difference in image presentation time: r(5) = −0.26, p = 0.57; all tests non-directional.) In addition, a 3-way between subjects ANOVA (multiple regression) was applied in order to attempt to model the difference in image presentation time observed between saccade and fixation conditions (for the 7 subjects with significant P3s) as a function of the corresponding differences between fixation and saccade conditions in P3 amplitude, start time, and persistence. There were insufficient degrees of freedom to model interactions, therefore only main effects were modeled. None of the 3 main effects was significant. (P3 amplitude not significant: F(1, 3) = 3.65, p = 0.15; P3 start time not significant: F(1, 3) = 2.27, p = 0.23; P3 persistence not significant: F(1, 3) = 3.47, p = 0.16).

Furthermore, three one-way within subject ANOVAs were applied to determine the effects of saccadic delay on the saccadic P3 parameters of amplitude, start time and persistence. A two-level factor of saccadic delay was created by calculating the median saccadic delay for each subject and then calculating the corresponding saccade P3 parameters for those trials in which the saccadic delay was greater than the median saccadic delay and those trials in which the saccadic delay was less than the median saccadic delay. For all three within subject ANOVAs, there were no significant effects of saccadic delay on saccade P3 parameters (effect of saccadic delay on saccade P3 amplitude not significant: F(1, 6) = 0.87, p = 0.39; effect of saccadic delay on saccade P3 start time not significant: F(1, 6) = 3.28, p = 0.12; effect of saccadic delay on saccade P3 persistence not significant: F(1, 6) = 1.96, p = 0.21). Note that the within subject ANOVAs were conducted without considering sphericity as correction can only result in higher p-values.

### Cluster Based Amplitude Comparisons of Fixation and Saccade P3s within Each Subject

Figure 3 is a plot of the target minus distracter waveforms at midline electrodes Fz, Cz and Pz for all subjects for the saccade and fixation conditions. Importantly, although the results in Figure 3 are shown only at midline electrodes, note that all results cited below were derived from statistical tests applied across all electrodes as discussed in detail in Methods. In 7 of the 8 subjects, the target minus mean distracter data displayed no significant differences with p<0.05 between saccade and fixation trials. The one exception was S6, who had a cluster of significant amplitude difference between the fixation and saccade P3s (shaded region in Figure 3) in which the saccade P3 exceeded the fixation P3 in amplitude, with an associated two-tailed false alarm probability of p = 0.001. Note that S6 was one of the two subjects that were not naïve regarding the goals of the experiment. It is therefore possible that the greater response in the saccade condition relative to the fixation condition observed in S6 could be due to attentional effects related to prior knowledge of the experiment.

Although it was not a significant difference, S5 had a cluster of saccade versus fixation amplitude positivity associated with a two tailed false alarm probability of p = 0.094. Note that, across the subjects, no consistent pattern of fixation P3 amplitude exceeding saccade P3 amplitude or vice versa existed. Furthermore, for all subjects other than S5 and S6, all clusters of amplitude differences between the saccade and fixation conditions had associated two-tailed false alarm probabilities of p>0.1.

### Comparing Fixation and Saccade P3 Grand Averages

Grand averages across all subjects of the TMD signal in the saccade and the fixation conditions are shown in Figure 4. The waveforms in Figure 4A are the grand averages at central midline electrodes Fz, Cz, Pz and Oz. Importantly, although the waveforms are shown in Figure 4A only at midline electrodes, note that all results cited below were derived from statistical tests applied across all electrodes as discussed in detail in Methods. It can be seen that the target minus distracter positivity was widespread in both the fixation and saccade conditions, and the maximum positivity shifted from frontal to posterior electrode sites in both the fixation and saccade conditions.

Non parametric tests as described in Methods revealed that there were no significant differences in start time and persistence between the fixation and saccade cross subject grand average (start time p = 0.75; persistence p = 0.81 two-tailed tests).

There were no significant clusters of saccade versus fixation amplitude difference in the grand average identified via non-parametric cluster statistics. However, there was a period in which the saccade P3 was greater than the fixation P3 associated with a maximum effect size of 1.58 from approximately 300–450 ms (this cluster is most visible in electrode Pz and Oz time courses in Figure 4A) The above cited effect size of 1.58 was the maximum effect size observed at electrode Pz in the 300–450 ms time period. In addition, there was a period in which the opposite effect was observed (in which the fixation P3 was greater than the saccade P3) from approximately 520 to 660 ms with a maximum effect size of −1.53 observed at electrode Cz in this time window.

Note again, however, that despite having substantial effect sizes these two clusters of amplitude difference did not reach significance. The saccade versus fixation positivity had an associated false alarm probability of p = 0.42 and the fixation versus saccade positivity had an associated false alarm probability of p = 0.27(two-tailed tests).

### Comparing Fixation and Saccade Target and Distracter Grand Averages Directly

The EPs associated with targets in the saccade and fixation conditions and the EPs associated with distracters in saccade and fixation conditions are compared in Figures 5 and 6, respectively. Importantly, although the waveforms in Figures 5A and 6A are shown only at midline electrodes, note that all results cited below were derived from statistical tests applied across all electrodes as discussed in detail in Methods. The z-score waveforms in Figures 5 and 6 were derived by first expressing the amplitude at each electrode and time point as a single sample t-statistic (mean over all trials as determined at each time point divided by standard error as determined across all trials at each time point). The t-statistic waveforms were then converted to z-score waveforms prior to cross-subject averaging. When the non-parametric methods for comparison described in Methods were applied to compare saccade and fixation target trials, a cluster in which the grand average saccade target waveform was significantly greater than the fixation target waveform was found from 0 to approximately 200 ms with an associated false alarm probability of p = 0.001 (Figure 5). In addition, a significant cluster of fixation target versus saccade target positivity was found from approximately 500 to 700 ms. Similarly, in the saccade distracter versus fixation distracter comparison shown in Figure 6, there was a significant cluster in which the grand average saccade distracter EP was greater than the grand average fixation distracter EP from approximately 0 to 200 ms with an associated false alarm probability of p = 0.008. In addition, there was a significant cluster in which the fixation distracter waveforms were greater than the saccade distracter waveforms with an associated false alarm probability of p = 0.023 and a temporal extent from approximately 300 to 650 ms.). The periods of significant difference observed in Figures 5 and 6 described above almost certainly were partially attributable to EOG components that were present in the saccade conditions but not in the fixation conditions. Note that saccades produce spike artifacts at saccade onset and step-like EOG artifacts [28] due to the rotation of the cornea-retinal dipoles during saccades. Furthermore, the differences observed in Figures 5 and 6 could be at least partially attributable to the saccadic lambda occipital response known to occur after saccades [15], [16].

Importantly, in contrast to the results shown in Figures 5 and 6, EOG and other saccade related EEG activity was expected to cancel out in the saccade target versus saccade distracter comparison shown in Figure 4. As was described in the previous section and as can be seen in Figure 4, no significant differences in the saccade and fixation P3 were observed. Due to the greater impact of EOG artifacts and other saccade-related EEG activity on Figures 5 and 6 relative to Figure 4, most of the conclusions pertaining to neural post-saccadic modulation that will be discussed in the following section rely upon the results of Figure 4 rather than Figures 5 and 6.

## Discussion

### Similarity of Saccade and Fixation P3s

P3 amplitude, persistence and start time are shown here to not differ significantly between fixation and saccade conditions when efforts are made to equate the stimuli in both conditions (see Methods for details). Although one subject did show a significant cluster of amplitude difference between the saccade and fixation P3s on the individual subject level (Figure 3), no significant amplitude differences were observed in the cross-subject grand average (Figure 4) and 7 out of 8 of the subjects did not exhibit significant differences between the saccade and fixation conditions on an individual subject level (Figure 3).

Both the saccadic and fixation P3 were widespread on the scalp, and showed a progression of TMD positivity that began at anterior scalp sites before shifting to posterior scalp sites. The shift in the maxima of the P3 from frontal to more posterior sites over time is consistent with past research investigating the neural sources of the P3. A joint fMRI/EEG study [37] and EEG studies [38] of the P3 indicate that a widespread network of brain regions is responsible for the P3 signal observed in EEG, with the target minus distracter positivity beginning in frontal regions and then progressing to more posterior regions.

### No Significant Modulations of the Saccadic P3

Importantly, in this study, in contrast to other electrophysiological studies in which post-saccadic amplitude modulations of neural activity were observed, rather than comparing post-saccadic neural activity directly to a baseline of neural activity recorded at fixation, we instead compared the difference of two post-saccadic signals (the saccade P3) to the difference of two signals measured at fixation (the fixation P3). Therefore, any general, saccade-related modulations in neural activity (including sensory responses and any saccade-related EOG artifacts) were expected to cancel out in the saccade target minus saccade distracter subtraction. Due to the removal of the common, saccade related EEG and EOG activity in the saccade target minus saccade distracter comparison, we were able to focus on neural amplitude differences between the saccade and fixation conditions associated with subjects’ categorization of the presented images. Indeed, it was found that when the saccade target and saccade distracter neural responses were compared directly to the corresponding fixation target and fixation distracter neural responses, there were significant amplitude differences in the EP waveforms (Figures 5 and 6). The significant amplitude differences between the saccade and fixation EPs when they were compared directly in Figures 5 and 6 likely are at least partially attributable to the post-saccadic amplitude modulations that have been observed in monkey electrophysiology [6], [7], but because the data herein are scalp EEG data it is not possible to distinguish any such post-saccadic amplitude differences from the multiple signals that are expected to be present in EEG data after any saccade but are not present at fixation, including EOG artifacts and neural activity such as the saccadic lambda occipital response [15], [16], [28].

As was seen in Figure 4, in contrast to Figures 5 and 6, when the saccade P3 was compared to the fixation P3 there were no significant differences observed. Importantly, this result does *not* contradict past work that has found post-saccadic modulations in visual responses after a saccade relative to visual responses measured at fixation. The results presented herein could be entirely consistent with past research if post-saccadic amplitude modulations affected the processing of saccade targets and saccade distracters equivalently. It has been found that amplitude modulations of visual saccadic responses relative to visual responses measured at fixation occur as early as the LGN [9]. Importantly, any amplitude modulations of post-saccadic responses relative to fixation responses originating in the LGN would be expected to alter saccade target and saccade distracter trials equivalently and cancel out in the saccade target minus saccade distracter subtraction. Therefore, the absence of an amplitude difference between the saccade and fixation P3 does not contradict saccade versus fixation amplitude modulations of visual responses originating in the LGN or other neural regions involved in the initial stages of visual processing prior to stimulus categorization. However, from the absence of a significant fixation and saccade P3 amplitude difference, it can be concluded that post-saccadic modulations are likely limited to sensory and other early neural responses after saccade landing, and that post-saccadic modulations have no or very limited effects on the P3.

In [25], a very early (140 ms post-saccadic landing) significant target minus distracter difference was observed after saccades in a free viewing condition. The early difference observed after saccades in [25] preceded the conventional fixation P3 by approximately 100 ms, and had a topography over central/parietal regions whereas the conventional fixation P3 generally begins at frontal electrodes [38]. Note that the post-saccade EPs in [25] were recorded in a free viewing condition in which it was relatively difficult to verify that target and distracter stimuli were indistinguishable to subjects prior to saccade landing. Therefore, one possible explanation of the results in [25] is that object recognition was not fully constrained to the time period after saccade landing, thereby resulting in an earlier, significant target minus distracter difference after saccade landing than at fixation. In contrast, in the experiments described here, saccades to the objects to be recognized were higher in amplitude (10°) as well as constrained in size and controlled for image visibility prior to saccade landing. Another, perhaps more critical difference between the experiments described here and those described in [25] is that, herein, target and distracter images were equated so that early sensory EPs were equivalent for targets and distracters and therefore canceled out in the target minus distracter subtraction [17]. In contrast, in [25] target and distracter images were not interchanged to balance out sensory differences between the two images. Therefore, it is possible that the very early, post-saccade target versus distracter difference observed in [25] resulted from post saccadic modulations of sensory EPs relative to fixation rather than post-saccadic modulations of the P3 or object recognition processes. The observations that the significant target minus distracter difference in [25] occurred very early after saccade landing and had a topography uncharacteristic of the P3 could also be indications that the post saccade difference observed in [25] was a post-saccadic modulation of sensory EPs relative to fixation rather than post-saccadic modulations of the P3 or of object recognition processes.

### Saccadic P3 and BCIs

The fact that the saccadic P3 was significant and reliably observed in all subjects that also had a significant fixation P3 indicates that the saccadic P3 is a robust signal that could be useful in BCIs. P3 start time, persistence and amplitude did not differ significantly in the saccade and fixation conditions. However, it should be noted that the P3 observed during natural eye movements could differ from the P3 observed here. Recall that the stimuli used herein were specifically designed to temporally restrict the moment of object recognition to occur after saccade landing. The rationale for allowing the target and distracter stimuli to only be distinguishable after saccadic landing was to restrict the time of object recognition in the saccade trials to the time period following saccadic landing. This restriction allowed for a direct comparison of the neural response time-locked to image presentation at fixation to the neural response time-locked to saccadic landing. In contrast, in natural viewing conditions, if object recognition is partly achieved before saccadic landing, the saccadic P3 might be expected to be more temporally diffuse than the saccadic P3 observed here. Development of a P3-based BCI allowing natural eye movements might therefore necessitate similar constraints in the timing of image visibility relative to saccade landing that were enforced here, and/or further investigation to determine whether or not the properties of the saccadic P3 are altered based upon the degree of visibility of images prior to saccade landing.

Another important aspect of the target and distracter stimuli was that they could be distinguished no matter where within the Gaussian envelopes the eyes landed, allowing for imprecision in eye position at saccadic landing without impacting the ability to distinguish targets from distracters. Therefore, subjects did not have to move their eyes after the initial saccadic landing to distinguish between target and distracter. A P3-based BCI allowing for natural eye movements would therefore need to employ similar constraints, and/or further investigation would be necessary to determine whether and how the saccadic P3 is altered if it is temporally distributed over the time course of multiple saccades. Given that humans move their eyes frequently, typically 2–3 times a second [1], [2], a P3-based BCI allowing for eye movements could provide users with an intuitive method of brain controlled computer interaction.
